# Avenues of research in dietary interventions to target tumor metabolism in osteosarcoma

**DOI:** 10.1186/s12967-021-03122-8

**Published:** 2021-10-29

**Authors:** Taiana Campos Leite, Rebecca Jean Watters, Kurt Richard Weiss, Giuseppe Intini

**Affiliations:** 1 Department of Oral and Craniofacial Sciences, University of Pittsburgh School of Dental Medicine, Pittsburgh, PA USA; 2 Center for Craniofacial Regeneration, University of Pittsburgh School of Dental Medicine, Pittsburgh, PA USA; 3 Department of Orthopaedic Surgery, University of Pittsburgh Medical Center, Pittsburgh, PA USA; 4 Department of Pharmacology & Chemical Biology, University of Pittsburgh School of Medicine, Pittsburgh, PA USA; 5 Hillman Cancer Center, University of Pittsburgh Medical Center, Pittsburgh, PA USA; 6 Department of Periodontics and Preventive Dentistry, University of Pittsburgh School of Dental Medicine, Pittsburgh, PA USA; 7 Department of Medicine, Division of Hematology and Oncology, University of Pittsburgh School of Medicine, Pittsburgh, PA USA; 8 McGowan Institute for Regenerative Medicine, University of Pittsburgh, Pittsburgh, PA USA

**Keywords:** Osteosarcoma, Tumor metabolism, Caloric restriction, Fasting, Ketogenic diet, Quercetin

## Abstract

Osteosarcoma (OS) is the most frequent primary bone cancer, affecting mostly children and adolescents. Although much progress has been made throughout the years towards treating primary OS, the 5-year survival rate for metastatic OS has remained at only 20% for the last 30 years. Therefore, more efficient treatments are needed. Recent studies have shown that tumor metabolism displays a unique behavior, and plays important roles in tumor growth and metastasis, making it an attractive potential target for novel therapies. While normal cells typically fuel the oxidative phosphorylation (OXPHOS) pathway with the products of glycolysis, cancer cells acquire a plastic metabolism, uncoupling these two pathways. This allows them to obtain building blocks for proliferation from glycolytic intermediates and ATP from OXPHOS. One way to target the metabolism of cancer cells is through dietary interventions. However, while some diets have shown anticancer effects against certain tumor types in preclinical studies, as of yet none have been tested to treat OS. Here we review the features of tumor metabolism, in general and about OS, and propose avenues of research in dietary intervention, discussing strategies that could potentially be effective to target OS metabolism.

## Osteosarcoma overview

Osteosarcoma (OS) is the most frequent primary bone cancer, although it is relatively rare in the general population, with 2–3 cases per million per year, accounting for 2% of pediatric cancers. There are two peaks of incidence—the first and highest is in children and adolescents, and the second one is in elderly (> 65 years)—, with a 1.4 to 1.0 male-to-female ratio, demonstrating male predominance. While the pediatric tumors are typically related to bone growth, the adult tumors are linked to Paget’s disease or radiation exposure. The most common locations are long bones, but other bones may develop OS, including the jaws and pelvis [1–6]. This tumor is characterized mainly by mesenchymal cancer cells that produce immature bone or osteoid. There are several histologic subtypes: osteoblastic, chondroblastic, fibroblastic, small cell, telangiectatic, high-grade surface, and extra-skeletal. They are further separated according to severity: high-grade (most aggressive), intermediate-grade, and low-grade (least aggressive). The most common type—around 85% of cases—is termed conventional OS: a high-grade tumor that develops in the intramedullary space (mostly osteoblastic subtype); while the other 15% correspond to intermediate-grade and low-grade OS, with periosteal (mostly chondroblastic subtype, located in the periosteum) and parosteal (mostly fibroblastic subtype, located on the surface of bones, over the periosteum) presentations, respectively [4, 5, 7]. OS, therefore, displays a very heterogeneous spectrum of phenotypes, reflecting an equally heterogeneous genetic configuration. Approximately 30–40% of patients with primary OS are expected to develop local relapse or metastases, the majority of which are to the lungs, accounting for most OS deaths [2, 8].

The etiology for OS remains elusive, and there is no single driver gene mutation responsible for its development; instead, numerous genetic alterations have been associated with OS [4, 6, 9]. Studies have shown that OS carries several chromosomal structural alterations, leading to mutations and even deletions or amplification of genes. Variations involving *TP53* and *RB1* genes are regarded as the leading causes for OS. In fact, transgenic murine models with conditional *p53* and *Rb1* knockouts have been shown to successfully develop spontaneous OS, modeling human osteosarcomagenesis [10, 11]. The rise of more modern and sensitive tools in molecular biology has allowed researchers to identify alterations in a plethora of genes related to different pathways in OS. Along with *TP53* and *RB1*, other commonly affected genes include *MYC*, *CDK4,* and *CDKN2* (cell cycle and apoptosis pathways); *AURKB* (mitosis pathways); *ATRX*, *BRCA1* and *BRCA2* (DNA damage repair); *PIK3CA*, *ALK*, *NF1* and *PTEN* (PI3K-mTOR/RAS signaling pathways), *NOTCH1-4* and *AKT1* (Notch signaling pathway); *DLG2* (Wnt signaling pathway); *SATB2* (osteoblastic differentiation); *VEGFA* (receptor tyrosine kinase pathway); and genes related to the IGF (insulin-like growth factor) signaling pathways [7, 12–20]. In addition to altered genes, OS also exhibits abnormal gene regulation and epigenetic mechanisms. For instance, dysfunctional long noncoding RNAs and microRNAs that modulate gene expression affect key OS features, such as apoptosis, cell cycle, proliferation, migration, invasion, and drug resistance [21–23]. Moreover, different DNA methylation patterns also seem to contribute with OS development, and higher methylation events were associated to more severe OS phenotypes [23–25]. This wide range of genetic and epigenetic variability renders OS as a very heterogeneous type of cancer, making it difficult to identify and develop novel therapies.

Researchers have proposed the existence of a specific cancer cell population responsible for cell renewal, metastasis, and drug resistance in cancers. These are cancer stem cells (CSCs), as they exhibit several hallmarks and markers in common with typical adult stem cells [26–28], and targeting their metabolism may represent a novel therapeutic strategy. The existence of these cells is a subject of controversy, due to lack of specific markers or standard identification strategies. However, the presence of CSCs has been consistently demonstrated in several cancers, such as leukemia, glioblastoma, breast, ovarian, colorectal, prostate, lung, liver, and kidney cancers [28–33], and it appears that CSCs are also present in OS. Specifically, OS CSCs share similarities with mesenchymal stem cells (MSCs), which points to a probable bone-marrow MSC and/or mesenchymal progenitor cell origin for OS CSCs [10, 11, 34–37]. Studies have demonstrated that OS CSCs have a constitutively-activated Wnt/β-catenin signaling pathway and overexpress the stemness-related genes *SOX2* and *KLF4*, and that CD133 can be used as a marker for OS CSC, as it is for other tumors [34, 38].

In general, the CSC phenotype exhibits high degrees of plasticity, with variations being determined by the tumor microenvironment (TME)—the stroma surrounding the growing tumor [27, 39]. Research has shown that the TME plays key roles in cancer survival and growth and is just as crucial to these processes as the cancer cells themselves [39, 40]. TME is characterized by being acidic and hypoxic—conditions that favor tumor growth and dissemination and that are shaped by the metabolic aberrancies of the cancer cells. The interplay between the tumor and its TME, which promotes tumor growth and metastasis, occurs through communication between TME-residing cells, CSCs, and bulk tumor cells, by means of secretion of high amounts of cytokines and growth factors, and through exosomes containing noncoding RNAs and other signaling molecules [39, 40]. All these features allow cancers to create an immunosuppressive environment, where host cells are recruited and “forced” into a phenotypic change favoring immune evasion. For instance, immune cells—particularly myeloid-derived suppressor cells (MDSCs), tumor-associated macrophages (TAMs) and tumor-infiltrating lymphocytes (TILs)—acquire anti-inflammatory and immunosuppressive phenotypes and are no longer capable of targeting cancer cells, while inhibiting the host’s effector cells [39–41]. Similar immunological mechanisms have been described in OS, which involve TAMs with an anti-inflammatory phenotype [14, 37, 42, 43]. The TME is also home to other cells—including MSCs, endothelial cells and cancer-associated fibroblasts (CAFs)—, all of which have been shown to play important parts in the OS TME [36, 42, 44–47]. A specific feature of the OS TME is the “vicious cycle” between osteoclasts and cancer cells, where the latter induce resorption of the extracellular matrix by the former, causing the release of embedded growth factors, which, in turn, promotes tumor growth [46, 48]. Furthermore, the OS TME was shown to reinforce immune evasion through overactivation of immunosuppressive pathways [49].

The standard treatment for OS includes neoadjuvant chemotherapy, which involves multiple cytotoxic agents, followed by surgical resection of the tumor—whether it be primary or secondary—, and adjuvant chemotherapy [2, 50]. Due to the aforementioned genetic heterogeneity and instability of OS, these therapies have thus far yielded unsatisfactory results, while the plasticity of OS CSCs has, as of yet, precluded the development of CSC-aimed treatments [2, 13, 15, 50, 51]. Other ongoing clinical trials employ immunotherapies, attempting to overcome the immunosuppressive features of OS tumors and their TMEs. Although much has been elucidated in regard to the molecular pathways involved, for instance PD-1 and PD-L1, none of the candidates has reached the expected success against OS [37, 49–52]. Currently, the standard treatment for patients diagnosed with localized OS sets the 5-year survival rate at around 70%. However, the 5-year survival rate for OS patients who have developed metastases has remained at 20% for the last 30 years, despite scientific progress [3, 8]. In light of this alarming figure, the development of more efficient treatments is desperately needed, especially those aimed at metastases, as these account for the vast majority of deaths.

Targeting tumor metabolism has emerged as a prominent field of study as a consequence of greater knowledge gained in recent years regarding the unique manner through which tumors metabolize nutrients in order to survive and proliferate [53–56]. By means of such targeting, the interplay between CSC and TEM may be disrupted, thus providing novel avenues of research in the field of OS treatment. Unfortunately, while a lot of studies exist about tumor metabolism in general, there is limited research that has been performed on possible targeting strategies for OS metabolism. We thus aim at reviewing the features of tumor metabolism, in general and about OS, and propose avenues of research in dietary intervention, discussing strategies that could potentially be effective to target OS metabolism.

## Tumor metabolism and osteosarcoma

**Fig. 1:**
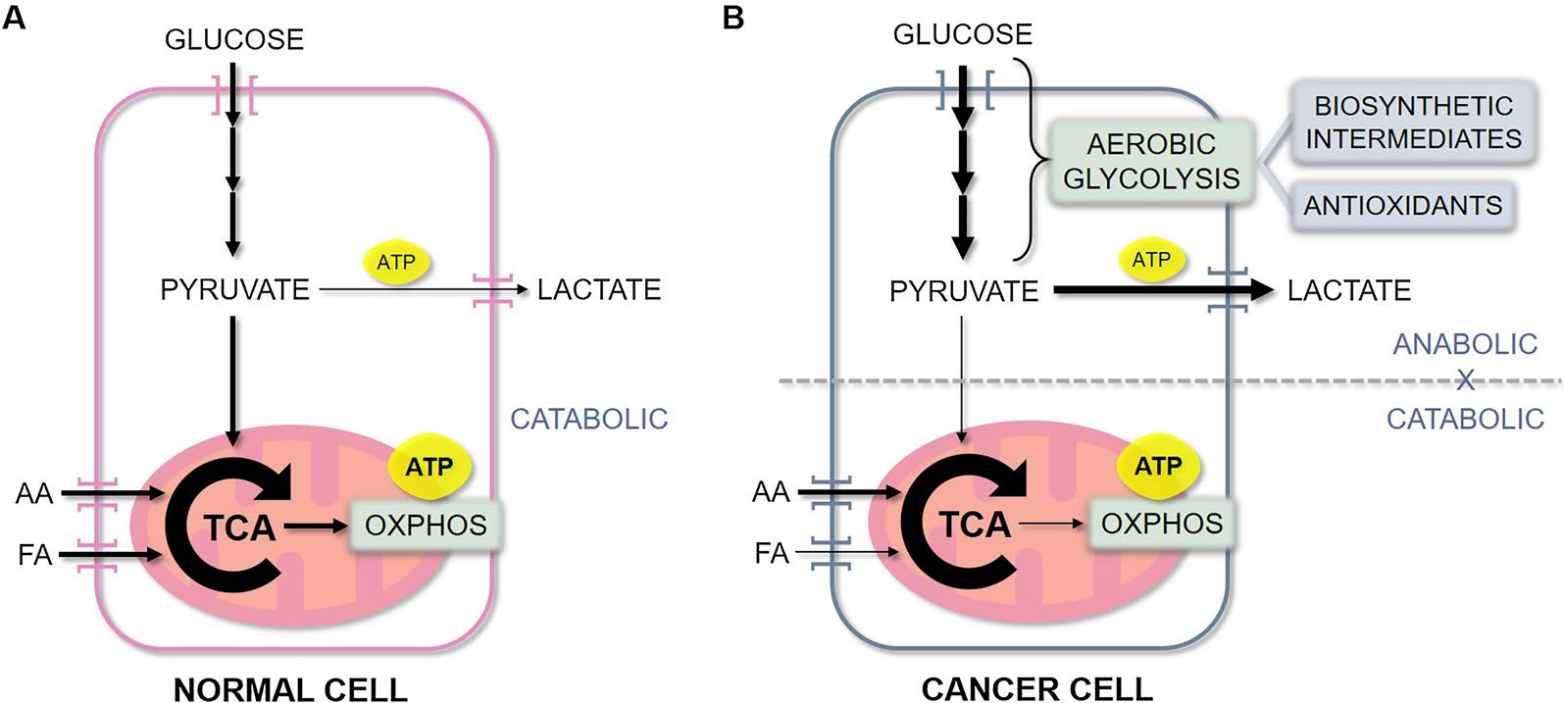
Energetic metabolism of normal cells versus cancer cells. **A** In normal cells, energy in the form of ATP is typically obtained from glucose through the coupling of its initial breakdown (yielding a small amount of ATP—2 ATPs per glucose molecule) with the oxidation of its products in the mitochondrial TCA cycle and OXPHOS (yielding the bulk of ATP—34 ATPs per glucose molecule). Alternatively, energy can also be obtained from fatty acids and amino acids, which are fueled into the TCA cycle and OXPHOS. **B** Cancer cells, on the other hand, uncouple the anabolic glycolytic pathway from the catabolic TCA cycle and OXPHOS. They increase glucose uptake, utilizing aerobic glycolysis as the main source of biosynthetic molecules, while producing antioxidants and high amounts of lactate. In parallel, these cells fuel the TCA cycle with amino acids and, to a lesser extent, fatty acids, allowing these cells to use OXPHOS as a means of obtaining the bulk of ATP, while uncoupled from glycolysis. AA: Amino acids; FA: Fatty acids; TCA: Tricarboxylic acid cycle; OXPHOS: Oxidative phosphorylation; ATP: Adenosine triphosphate

Over the last decade, we have acquired a deeper understanding of the particular features of cancer cell bioenergetics. In fact, the deregulation of cellular energetics was included among the eight hallmarks of cancer in 2011 [57]. In normal cells, energy is typically obtained from glucose through the coupling of its initial breakdown (glycolysis) with the oxidation of its products in the mitochondrial oxidative phosphorylation (OXPHOS) (Fig. 1A). On the other hand, most cancer cells uncouple these two pathways and display metabolic plasticity, depending on nutrient and oxygen availabilities. Cancer cells typically utilize aerobic glycolysis as their preferred method of rapidly obtaining intermediate molecules, which serve as building blocks for their anabolic state, while producing antioxidants and high amounts of lactate [53, 58, 59]. Indeed, research shows that high cellular glucose uptake occurs in several types of cancer, including OS [60, 61], and in many cases it is associated with tumor aggressiveness [58, 61–63]. In parallel, these cells fuel the tricarboxylic acid (TCA) cycle in the mitochondria with other nutrients, especially amino acids (mostly glutamine), and the intermediates from this pathway also provide precursors for biosynthesis. This allows these cells to use OXPHOS as a means of obtaining ATP, while uncoupled from glycolysis, thus maintaining high proliferative rates [56, 58, 59] (Fig. 1B). This metabolic plasticity is especially evident among CSCs as part of their diverse phenotypes, providing them with the ability of adapting to stresses, such as nutrient deprivation, hypoxia, and the presence of antitumor drugs [64, 65]. While these features of cancer cell bioenergetics seem to apply to OS as well, the exact characteristics of the OS metabolism remain to be elucidated. For instance, studies have shown that, although metabolically plastic, OS CSCs follow this trend and preferably utilize the glycolytic pathway, with downregulation of the TCA cycle and OXPHOS pathways [66, 67].

It is important to note that the metabolic reprogramming observed in tumor cells is not a passive event, nor a mere consequence of tumor development. Rather, it is a deliberate and required process, where tumor growth-related pathways and cellular metabolism are intimately connected [56]. For example, mutations in the TCA cycle enzymes isocitrate dehydrogenases-1 and -2 can directly contribute to tumorigenesis in several cancers, including glioma, OS, and chondrosarcoma, by altering the function of other enzymes, which either degrade pro-tumorigenic factors or regulate methylation of histones or DNA [68, 69]. Other metabolism-related pathways are direct targets of oncogene and defective tumor suppressor gene products. For example, the PI3K/Akt-mTOR pathway, which is typically altered in cancers—including OS—, upregulates cellular glucose uptake, while oncogenes *c-MYC* and hypoxia-inducible factor-1 (*HIF-1*) upregulate glycolytic enzymes [59, 70–72]. Increased levels of insulin-like growth factor-1 (IGF-1) and its receptor generally occur in OS and in other cancers, and also activate the PI3K/Akt-mTOR pathway, promoting tumor growth [73–75].

**Fig. 2:**
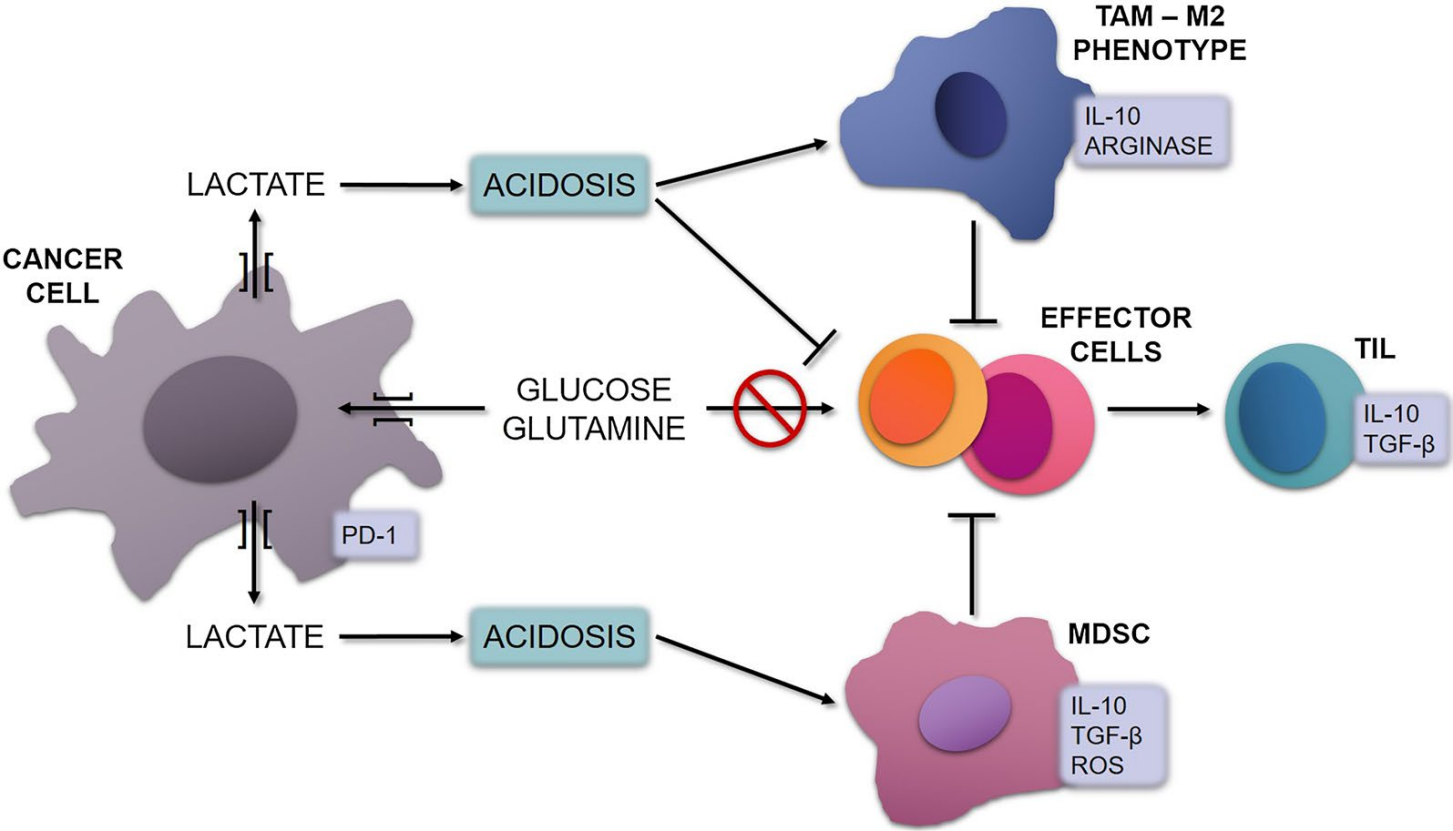
Cancer cell metabolism induces immunosuppression in the TME. Cancer cells compete with the host’s effector cells for nutrients. Their dysregulated metabolism leads to increased uptake of glucose and glutamine, depleting the effector cells from these nutrients, thus hindering their activity. Additionally, cancer cells release high amounts of lactate into the extracellular space, resulting in acidosis. This, in turn, makes the TME favorable for TILs, TAMs and MDSCs, and unsuitable for effector cells. TAMs are induced to express an M2 anti-inflammatory phenotype, secreting Il-10 and arginase. TILs also exhibit an anti-inflammatory phenotype, secreting Il-10 and TGF-β, while MDSC secrete Il-10, TGF-β and ROS. These factors further inhibit effector cells, promoting immunosuppression. TAM: Tumor-associated macrophages; IL-10: Interleukin-10; TGF-β: Transforming growth factor-β; ROS: Reactive oxygen species

The metabolic reprogramming that occurs in cancers in general, and in OS as well [43], also influences the host’s immune response, representing a key contributor to the creation of an immunosuppressive TME [40, 41]. The host’s effector cells have similar metabolic requirements as tumor cells, and thus compete with them for nutrients in the TME, especially glucose and glutamine. This competition for nutrients, together with the lactate-induced acidosis, make the TME favorable for TILs, TAMs and MDSCs, and unsuitable for effector cells, thus promoting immune evasion. TILs and TAMs have anti-inflammatory phenotypes and either secrete or induce factors that promote immunosuppression and tumor invasion, such as interleukin-10 (Il-10), TGF-β, PD-1, reactive oxygen species (ROS) and arginase [40, 43, 49, 65, 76–80] (Fig. 2).

Given the clear importance of tumor cell bioenergetics, various cancer metabolism-targeting strategies—consisting of drugs directed at specific metabolic components and pathways—have been explored in both preclinical and clinical studies over the last several years [55, 81, 82]. The most studied are the ones targeting enzymes involved in the glycolytic pathways, such as 2-deoxy-D-glucose, oleanolic acid, and 3-bromopyruvate, as reviewed in [53], 55. Another important group includes inhibitors of transporter molecules, especially those involved in glucose and glutamine uptake, as well as lactate transport, reviewed in [55]. Drugs interfering in pathways that regulate tumor cell metabolism are also being studied, including c-MYC, mTOR and PI3K/Akt inhibitors, and AMPK activators, reviewed in [82]. A few of these studies were carried out on OS. For example, preclinical investigations have shown that metformin—an AMPK inducer, which inhibits OXPHOS and causes oxidative stress—was capable of inducing apoptosis of OS cell lines, as well as preventing tumor growth in xenograft OS mouse models [83, 84]. In another study, a glutaminase inhibitor was utilized in combination with metformin in a xenograft OS mouse model, and the combination was more successful than treatments alone and non-treated controls in inhibiting primary tumor growth, as well as in preventing metastases [85]. Furthermore, glycolytic activities were reduced in OS cell lines and in a xenograft OS mouse model with the use of a synthetic oleanolic acid derivative—which inhibits pyruvate kinase, a glycolytic enzyme—, resulting in tumor growth inhibition [86]. However, despite reaching pre-clinical success, most of the proposed therapies have not thus far been approved as anticancer drugs for humans.

A controlled diet-based approach may represent an attractive and inexpensive alternative to target the metabolism in cancer that clinicians could use in combination with existing treatment regimens for their patients. Of course, patients should avoid adopting diet regimens without doctors’ supervision. The most studied diet-based approaches to treat cancer are: caloric restriction, fasting, and the ketogenic diet, and the use of dietary supplements. However, thus far, these diets have not been analyzed in an OS context. The following section will review what investigators have uncovered on the use of these dietary approaches as potential cancer treatments in general, proposing to promote these avenues of research in the field of OS.

## Dietary interventions that may target osteosarcoma

### Caloric restriction diet

The caloric restriction (CR) diet typically refers to a continuous reduction of normal daily recommended calorie intake by approximately 30%, with proportional reduction of all macronutrients, while maintaining adequate amounts of vitamins and minerals, without developing malnutrition. It has been widely studied and applied to prevent or reverse obesity and aging [73, 87, 88]. However, there are concerns regarding patient tolerance to this diet, especially considering that it causes weight loss and cancer patients may be prone to developing cachexia. Therefore, clinical studies that utilize this diet in cancer patients always include careful nutritional monitoring [54, 87].

The main mechanism of action for its antitumor effects is through inhibition of IGF-1. As a result, the IGF-1/PI3K/Akt signaling pathways are repressed, thus inducing apoptosis and reducing angiogenesis, cell cycle progression, and metastasis [73, 89, 90]. Additionally, AMP-activated protein kinase (AMPK) is activated, which, in turn, also inhibits the PI3K/Akt-mTOR pathway, and, consequently, the anabolic pathways [87].

Although dietary interventions against cancer have not been extensively explored, the antitumor effects of CR have been confirmed in a number of preclinical investigations—none, however, for OS. In mice models for cancer, colon cancers showed reduced tumor volumes when compared to controls [91], while prostate [92], breast [93] and pancreatic [94] cancers were prevented. In a rat model for breast cancer, not only did CR prevent tumor development, but it also prevented its progression, with the demonstration of an inhibitory effect on CSCs [95]. In addition, CR has been studied as an adjuvant to standard chemotherapy and radiation therapy, demonstrating the ability to improve treatment results. The addition of CR to ganitumab (an anti-IGF-1R drug) improved tumor reduction in prostate cancer mice when compared to the drug alone, with increased apoptosis and reduced cell proliferation [96]. In a triple-negative breast cancer mouse model, CR reversed treatment-induced inflammation from cisplatin through IGF-1 modulation, thereby reducing drug resistance [97]. CR was also shown to improve radiation therapy treatment in two aggressive breast cancer models, delaying metastasis and tumor growth, and increasing survival in comparison to the radiation therapy alone [98]. Another study using a murine triple-negative breast cancer model demonstrated that CR was capable of decreasing the number of intratumoral TILs and increasing the number of effector cells after radiation therapy [99].

Given the increased levels of IGF-1 and the strongly activated PI3K/Akt-mTOR pathway observed in OS [60, 72, 75], CR could potentially be effective against this tumor. It would be, therefore, interesting to study the effects of CR on development and metastasis of OS.

### Fasting diet

Fasting is a complete deprivation of nutrients during a specific period of time, with no food intake—only water. However, there are different versions of clinically-feasible fasting. The most common are the fast-mimicking diets (FMDs), which can be performed with varied protocols. For instance, FMDs can be followed by means of cycles of restricted access to food (very low caloric intake—typically 300 to 1100 kcal a day—, for example, five consecutive days once a month, for 3 months) [100], or by means of intermittent fasting (IF), which involves a specific number of hours of complete nutrient deprivation cycled with a period of unrestricted access to food (for example, 16–18 h daily) [100–102].

The rationale for using fasting as an antitumor therapy is to limit the amount of glucose and amino acids (AA) available to cancer cells, thereby disturbing their metabolism. Mechanistically, the effects of fasting regimes seem to be similar to those of CR, in that IGF-1 is reduced and AMPK is activated, inhibiting the PI3K/Akt-mTOR pathway. Additionally, due to the restriction of glucose, aerobic glycolysis, on which tumors rely for proliferation and immunosuppression, is also inhibited [17, 101, 103]. Moreover, the limitation of AA availability also impacts cancers, given that in general, they require large amounts of AA for cell proliferation and survival. Although they are capable of synthesizing non-essential AA intracellularly, they still require exogenous sources of both essential and non-essential AA, as the produced amount is insufficient for the cell requirements [104, 105]. Therefore, disturbing AA metabolism is yet another mechanism of action of fasting.

Fasting has successfully inhibited cancer cells in a few preclinical studies, although none of these studies focused on OS. For instance, an astrocytoma mouse model received an IF protocol (24 h in alternate days) and exhibited significantly smaller tumors with significantly higher survival rates, relative to controls [106]. Similarly, a colorectal cancer mouse model received an IF regime and showed significantly slowed tumor growth. Interestingly, the authors verified that the diet suppressed M2 polarization of TAMs, pointing to a more favorable immune response switching [107]. On the other hand, IF (24 h, twice a week) did not exert antitumor effects on a prostate cancer mouse model [108]. A colorectal cancer mouse model was also used to evaluate the effect of an FMD, with two weekly cycles, where each week was divided into three parts: day 1 with 50% of normal caloric intake, days 2–3 with 10% of normal caloric intake, and days 4–7 with normal food intake. The results showed, once again, a slower progression, with inhibition of proliferation and demonstration of a significantly reduced glucose intake [109]. Another study demonstrated that two weekly cycles of two-day fasting regimes inhibited colon cancer growth in mice in comparison to controls, while also demonstrating that cells switched from a glycolysis-based metabolism to an oxidative phosphorylation-based metabolism. In this same study, the authors also tested the effect of oxaliplatin—a standard chemotherapeutic agent—alone and in combination with fasting and verified that the combination therapy had the best results [103]. Other studies also evaluated fasting in combination with other treatments. For instance, in a lung cancer mouse model, animals underwent two cycles of 48 h-fasting and one cycle of 24 h-fasting in combination with a PD-1 immune checkpoint blockade. Results indicated that the combination treatment had a synergistic effect, inhibiting tumor progression and metastasis more effectively than untreated controls or either diet or anti-PD-1 alone [110].

Importantly, short-term fasting (48–60 h pre- and post-chemotherapy or radiation therapy) was demonstrated to induce differential stress resistance both in animal and human studies. By means of this mechanism, normal cells acquire a protected and slow-division state against the toxic effects of chemotherapy or radiation therapy, or other toxic agents, while cancer cells remain susceptible. Based on this lower toxicity to the host’s cells, in many instances, fasting allows the employment of higher and, thus, more effective, treatment doses [101, 111–116].

As mentioned, studies about the effects of fasting on development and treatment for OS are missing. Yet, given that OS relies heavily on glycolysis, as well as on the IGF-1/PI3K/AKT signaling pathways [60, 66, 67, 75], fasting approaches could also be a promising tool against OS. Moreover, the metabolism of many AA was shown to be upregulated in OS, especially alanine, aspartate, glutamate, arginine, proline, cysteine and methionine [66]. Fasting could, therefore, also impact AA metabolism in OS cells, contributing to the other anticancer effects. Future preclinical studies are needed to evaluate the effectiveness of this approach for treatment of OS.

### Ketogenic diet

The ketogenic diet (KD) is defined as a high-fat, moderate-protein, and low-carbohydrate diet, typically with a 4:1 ratio of fat to protein and carbohydrates. Systemically, the low glucose concentrations inhibit insulin and activate glucagon secretion, and the body is forced into a ketogenic state, whereby liver cells oxidize fatty acids, producing ketone bodies—namely acetoacetate, β-hydroxybutyrate and acetone. These enter the circulation and are distributed to other tissues, serving as fuel for cell metabolism, after entering the TCA cycle. This diet was initially formulated to treat epilepsy and is currently also being used to treat obesity [117–119]. The KD is usually well-tolerated, although it may lead to mild side-effects, such as lethargy and nausea; however, if adequately monitored, they are easily avoided [54, 120, 121].

The very low amount of glucose ingestion due to KD blocks the cells’ ability—including cancer cells’—of using the glycolytic pathway. This hinders the cancer’s main source of building blocks for proliferation, thus leading to a lower proliferative rate. The limitation of the glycolytic pathway also impacts one of the most important sources of antioxidants, leading to higher oxidative stress, as cancer cells produce high amounts of ROS, thus potentially causing apoptosis [117, 122, 123]. Similar to the previously mentioned diets, the KD is also capable of reducing IGF-1 levels, which induces activation of AMPK, inhibiting the PI3K/Akt-mTOR pathway [119, 124].

A possible limitation of reducing glucose intake is the fact that it could further hamper the host’s defense, as effector cells require glucose to initiate an immunologic response. However, it has been demonstrated that effector cells are still able to mount an immune response despite low amounts of glucose. Although the reason behind this is unknown, it was hypothesized that these cells could utilize ketone bodies instead of glucose and still maintain their metabolic activities [125].

Compared to CR and fasting, there are more animal studies on the antitumor effects of KD, especially for brain tumors. However, none of these studies were performed on OS. Because of the blood–brain barrier, brain tissue cannot metabolize fatty acids; thus, the brain relies on glucose as its main source of energy. In the absence of glucose, brain cells are able to metabolize ketone bodies, while brain tumors likely have defective ketone metabolisms and rely mostly on a glycolytic metabolism, therefore rendering them more sensitive to a KD [126, 127]. In glioblastoma mouse models, KD significantly slowed tumor progression and increased survival rates when compared to normal diet [125, 128, 129]. Another study using a glioma mouse model interestingly found that the KD was able to influence the TME, by downregulating hypoxia-related markers, which favor tumor progression, in addition to downregulating angiogenesis-related factors [130]. KD was effective in reducing tumor growth and improving survival rates in neuroblastoma mice [131]; however, it was unsuccessful in eliciting antitumor effects in medulloblastoma mice [132]. Lussier et al. analyzed the immunologic effects of the KD’s impact on the TME of gliomas in a mouse model and found that it was able to promote phenotypic changes in the TILs. Tumors treated with KD showed an increased CD4+ T cell population and a reduced regulatory T cell population when compared to controls, while reducing expression of PD-1 on CD8+ T cells, thus opposing immunosuppression [125]. The KD produced significantly smaller tumors in a squamous cell carcinoma mouse model [133], as well as in breast [123, 134], prostate [135], colorectal [136, 137], pancreatic [80], and gastric [138] cancer mouse models, relative to controls. While a KD also produced these antitumor effects in a liver cancer mouse model [139], it failed to do so in another study by the same group, where the dietary intervention took place when tumors were in a later stage, suggesting that the KD, in this case, had a more preventive than therapeutic action [140]. The beneficial effects of KD may also be cancer-specific, since studies on a mouse model of melanoma showed that KD enhanced tumor growth [141]. In other studies, KD was evaluated in combination with other treatments. For instance, KD synergized with radiation therapy [142] and chemotherapy [143, 144], leading to significantly smaller gliomas and prolonged survival in mice in comparison to stand-alone treatments and controls. Similar effects were observed in neuroblastoma [145] and breast cancer [146] mouse models treated with KD and chemotherapy, as well as in a lung cancer mouse model treated with KD, chemotherapy and radiation therapy [147].

Fewer studies analyzing the effects of KD have been conducted in humans, most of which had the primary focus of determining feasibility and safety. These studies concluded that KD is overall safe and feasible for different cancer patients, in most cases improving quality of life; however, none showed antitumor effects of KD as standalone treatment [148–156]. An interesting find was a significant decline in the amount of lactate in head and neck cancers of patients treated with KD compared to those with normal diets, which may confirm the potential to counteract immunosuppression [157].

As with the aforementioned diets, the KD has yet to have its antitumor effects tested on OS. However, it does have potential to be effective in this regard, as the reduced amount of glucose impacts the glycolytic pathways, as well as the IGF-1/PI3K/AKT signaling pathways, which have been shown to be enhanced in OS [60, 66, 67, 75]. It could, therefore, be interesting to develop new preclinical studies to test this diet on OS.

### Dietary supplements: quercetin

Dietary supplements could also be used in addition to, or in alternative to, dietary modifications [158, 159]. However, no dietary supplements have been approved as anticancer therapies in the United States. This is due, in part, to the observational nature of the available studies about dietary supplements, as well as the lack of regulation for their use [159, 160]. Among the various dietary supplements available, quercetin—largely used worldwide and found in fruits, vegetables, tea, and wine—has shown some potential. Quercetin is a bioactive flavonoid with antioxidant, antiestrogenic, and antiproliferative effects that may be at the basis of its therapeutic properties [161]. Several in vitro and in vivo preclinical studies have been conducted regarding its anticancer effects, as reviewed in [162, 163] and shown in [164]. Although the exact mechanisms of Quercetin anticancer properties are not clear, they seem to occur via modulation of VEGF, apoptosis, PI3K/Akt/mTOR, and Wnt/β-catenin signaling pathways [162, 163]—all of which are affected in OS. Indeed, quercetin has consistently shown effectiveness against OS in vitro and in vivo. For instance, studies have shown that Quercetin induces inhibition of proliferation, cell cycle arrest, and apoptosis of MG-63 [165, 166], U2OS, Saos-2 [166], HOS [167], and 143B [168] cells. Additionally, Wu et al. showed that quercetin induces autophagy of MG-63 cells in vitro and in vivo [169]. Lan et al. demonstrated that quercetin attenuated cell migration and invasion, with downregulation of HIF-1α, VEGF, MMP2, and MMP9 expression on HOS and MG-63 cells, when compared to vehicle control. They also verified reduction in the lung metastases in OS xenograft models [170]. While these studies indicate that Quercetin may have some effects on OS, additional preclinical studies and randomized clinical trials are needed to confirm its anticancer efficacy. Considering the significantly high doses, well above the normal dietary intake, that may be required to achieve the anticancer effects, safety considerations should be carefully assessed [171, 172].

## Conclusions and future perspectives

Considering the importance of the aberrant bioenergetics of cancer to its progression and aggressiveness, targeting tumor metabolism may represent a promising strategy to treat cancer in general, and may represent an interesting avenue of research as a novel approach to treat OS. Although, to date, dietary approaches have not been evaluated as treatments for OS, it would be interesting to initiate a series of preclinical and clinical investigations to explore their impact on OS cells, uncovering possible mechanisms of action and novel therapeutic strategies. These studies should aim at testing the effects of different diets or diet supplements on CSCs metabolism and their interplay with the TME. Although CR, fasting and ketogenic diets, and the supplemental use of quercetin are overall well-tolerated by patients [100, 101, 155, 171, 173], it would be necessary to verify safety along with efficacy.

Moreover, human diets in general appear to be more effective when used in combination with other anticancer treatments [54, 101, 174]. One of the reasons why tumors are resistant to anticancer therapies is the fact that they exhibit multiple redundant pathways to promote growth, survival, and metastasis, which renders single-target therapies ineffective. This holds especially true for OS, which is highly heterogeneous genetically and phenotypically. Therefore, the key to improving success in OS could be the use of combination therapies: a dietary approach could likely sensitize OS cells to another antitumor therapy. For example, a recent study in pediatric patients with acute lymphoblastic leukemia showed improvement of response to chemotherapy when combined with CR [175].

In conclusion, it would be interesting to include dietary interventions as both stand-alone or combination therapies as subject of future avenues of research in OS. Very little is known at the moment, and therefore extensive preclinical in vitro and in vivo investigations should be performed prior to clinical trials.

## Data Availability

Not applicable.

## Abbreviations

OS: Osteosarcoma
OXPHOS: Oxidative phosphorylation
IGF: Insulin-like growth factor
CSC: Cancer stem cell
MSC: Mesenchymal stem cell
TME: Tumor microenvironment
MDSC: Myeloid-derived suppressor cell
TAM: Tumor-associated macrophage
TIL: Tumor-infiltrating lymphocyte
CAF: Cancer-associated fibroblast
PD-1: Programmed cell death protein-1
PD-L1: Programmed cell death protein-1 ligand
IDO: Indoleamine dioxygenase
TGF-β: Transforming growth factor-beta
STAT3: Signal transducer and activator of transcription 3
VEGF: Vascular endothelial growth factor
IL-10: Interleukin 10
TCA: Tricarboxylic acid
AA: Amino acid
FA: Fatty acid
HIF-1: Hypoxia-inducible factor-1
ROS: Reactive oxygen species
CR: Caloric restriction
AMPK: AMP-activated protein kinase
IGF-1R: Insulin-like growth factor receptor
FMD: Fasting-mimicking diet
IF: Intermittent fasting
KD: Ketogenic diet
